# Healthy lifestyle decreases the risk of the first incidence of non-communicable chronic disease and its progression to multimorbidity and its mediating roles of metabolic components: a prospective cohort study in China

**DOI:** 10.1016/j.jnha.2024.100164

**Published:** 2024-02-01

**Authors:** Chong Lai, Ruiyi Fu, Changzhen Huang, Lu Wang, Haiqing Ren, Yimin Zhu, Xuhui Zhang

**Affiliations:** a Department of Urology, the First Affiliated Hospital, Zhejiang University School of Medicine, Hangzhou, China; b Department of Epidemiology & Biostatistics, School of Public Health, Zhejiang University, Hangzhou 310058, Zhejiang, China; c Dongyang Traditional Chinese Medicine Hospital, Dong Yang, Zhejiang, People’s Republic of China; d Basic Discipline of Chinese and Western Integrative, School of Public Health, Zhejiang Chinese Medical University, Hangzhou, 310053, Zhejiang, China; e Hangzhou Center for Disease Control and Prevention, Hangzhou, 310051, Zhejiang, China

**Keywords:** Healthy lifestyle, Non-communicable chronic diseases (NCDs), Multiple chronic conditions (MCCs)

## Abstract

**Objectives:**

To identify the influence of healthy lifestyles on the incidence of the first NCD (FNCD), multiple chronic conditions (MCCs), and the progression from FNCD to MCCs. *Design: cohort study. Setting: Zhejiang, China*

**Participants:**

10566 subjects (55.5 ± 13.5 years, 43.1% male) free of NCDs at baseline from the Zhejiang Metabolic Syndrome prospective cohort.

**Measurements:**

Healthy lifestyle score (HLS) was developed by 6 common healthy lifestyle factors as smoking, alcohol drinking, physical activity, body mass index (BMI) and waist-to-hip ratio (WHR). Healthy lifestyle data and metabolic biomarkers collected via a face-to-face questionnaire-based interview, clinical health examination and routine biochemical determination. Biochemical variables were determined using biochemical auto-analyzer. Participants were stratified into four group based on the levels of HLS as ≤2, 3, 4 and ≥5. Multiple Cox proportional hazards model was applied to examine the relationship between HLS and the risk of FNCD, MCCs and the progression from FNCD to MCCs. The population-attributable fractions (PAF) were used to assess the attributable role of HLS. Mediating effect was examined by mediation package in R.

**Results:**

After a median of 9.92 years of follow-up, 1572 participants (14.9%) developed FNCD, and 149 (1.4%) developed MCCs. In the fully adjusted model, the higher HLS group (≥5) was associated with lower risk of FNCD (HR = 0.68 and 95% CI: 0.56−0.82), MCCs (HR = 0.31 and 95%CI: 0.14−0.69); and the progression from FNCD to MCCs (HR = 0.39 and 95%CI: 0.18−0.85). Metabolic components (TC, TG, HDL-C, LDC-C, FPG, and UA) played the mediating roles with the proportion ranging from 5.02% to 22.2% for FNCD and 5.94% to 20.1% for MCCs. PAFs (95%CI) for poor adherence to the overall healthy lifestyle (HLS ≤ 3) were 17.5% (11.2%, 23.7%) for FNCD, 42.9% (23.4%, 61.0%) for MCCs, and 37.0% (15.5%, 56.3%) for the progression from FNCD to MCCs.

**Conclusions:**

High HLS decreases the risk of FNCD, MCCs, and the progression from FNCD to MCCs. These effects are partially mediated by metabolic components. Maintaining healthy lifestyles might reduce the disease burden of common chronic diseases.

## Graphical abstract

The healthy lifestyle score is associated with the risks of FNCD, MCCs and the progression from FNCD to MCCs.
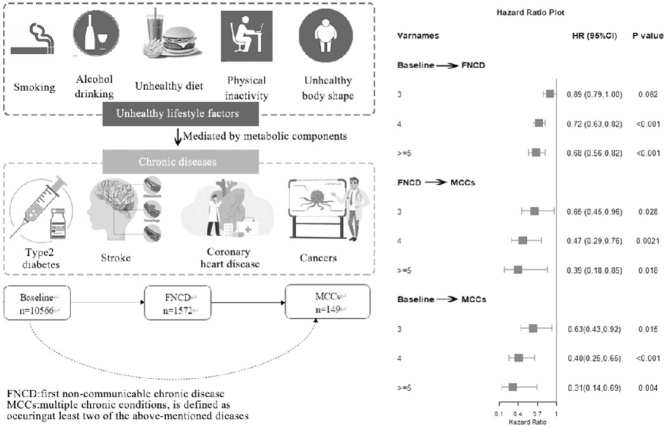

## 1 Introduction

The aging population and the widespread prevalence of health risk factors drive a rise in the burden of non-communicable diseases (NCDs). Meanwhile, advances in medicine, improvement of sanitary conditions, and popularization of the social pension insurance system play important roles in lengthening life expectancy. As a result, a growing population is living with non-fatal chronic conditions. The number of residents with chronic diseases is gradually increasing. Cancer, type 2 diabetes mellitus (T2DM), and cardiovascular diseases (CVD) are particularly relevant as they are the most common NCDs and represent major causes of morbidity, disability, and impaired quality of life [1]. Moreover, a large proportion of chronic patients suffer from two or more chronic diseases simultaneously, and they are referred to as having multiple chronic conditions (MCCs) [2,3], which has a prevalence of over 50% in the Chinese older population [4,5]. The co-morbidity of MCCs has a huge burden on both the health service utilization and costs, increases the risk of disability, and reduces the quality of life in older adults [[6], [7], [8]]. Thus MCCs might cause more adverse health consequences than having a single chronic disease, both physically and mentally [9].

According to studies of the China Kadoorie Biobank cohort and the China PEACE Million Persons Project, adherence to a healthy lifestyle was associated with substantially lower risks of all-cause, cardiovascular, respiratory, and cancer mortality in Chinese adults [10,11]. Low-risk lifestyle habits reduced the single morbidity of T2DM and CVD [12,13]. Han et al. revealed that healthy lifestyle behaviors were negatively associated with cardiometabolic multimorbidity [14]. Promoting healthy lifestyles could be associated with gains in disease-free life expectancy in the Chinese population [15]. In European countries, it has been shown that pre-diagnostic healthy lifestyle behaviors were inversely associated with the risk of cancer and cardiometabolic diseases with the prognosis of these diseases by reducing the risk of multimorbidity [16]. However, it remains unclear how decreasing the adoption of major healthy lifestyle behaviors through public health interventions helps achieve the MCCs in the Chinese population.

To expose the potential association between overall lifestyle behaviors on the incidence of FNCD and its progression towards MCCs, we analyzed the risks of FNCD, the progression from FNCD to MCCs and MCCs in different levels of healthy lifestyle score (HLS), which was developed with six lifestyle factors of cigarette smoking, alcohol drinking, physical activity, diet behavior, and obesity. We also comprehensively evaluated the effect of a series of metabolic components of lipids, fasting plasma glucose, and serum uric acid on mediating the relationship between healthy lifestyle behaviors with FNCD and MCCs. Population-attributable fractions (PAF) and preventable incidence of non-communicable disease were also evaluated.

## 2 Materials and methods

### 2.1 Study population

All subjects (N = 15,946) recruited in this study were from the Zhejiang Metabolic Syndrome prospective cohort. The cohort was followed up from the baseline investigation in January 2009 to June 2022. Details of the cohort were described in a previous publication [17]. The exclusion criteria of the subjects for the current study were as follows: (1) prevalence of NCDs at baseline, including type 2 diabetes mellitus (T2DM), cancer, and CVD (including stroke and coronary heart disease (CHD)) (n = 1165); (2) with missing data of lifestyle factors or the outcome variables (n = 4156); (3) lost to follow-up or have abnormal survival time (n = 59). Meeting with the above conditions, a total of 10,566 participants were finally selected at baseline. The study was conducted under the guidance of the Declaration of Helsinki. All procedures involving human subjects were approved by the institutional review board at the Zhejiang University School of Public Health. Written informed consent was obtained from all participants.

### 2.2 Definition of lifestyle factors and development of HLS

Each participant underwent a face-to-face questionnaire-based interview, clinical health examination and routine biochemical determination [17]. Based on previous studies and Dietary Guidelines for Chinese Residents, six lifestyle factors (smoking, alcohol intake, physical activity, dietary habits, body mass index (BMI), and waist-to-hip ratio (WHR)) were taken into analysis [10,18]. Healthy lifestyles included: (1) nonsmoking; (2) none to moderate alcohol drinking, which was defined as never or less than two times of alcohol intake per week. (3) high physical activity level, defined as the highest quartile of energy expended for each activity in metabolic equivalent (MET) hours per week (MET-h/week). The International Physical Activity Questionnaire (IPAQ) was used for the assessment of the average amount of time per week engaged in exercise activities [19]. (4) healthy dietary habits, defined as meeting at least 2 ideal diet components which were classified as top quartile consumption of fruits, vegetables, and lowest quartile consumption of red meat. We scored the subjects based on the consumption of 3 dietary components (fruits, vegetables and red meat), ranging from 0 to 3. (5) none general obesity, defined by BMI (18.5 ≤ BMI < 24.0 kg/m^2^) [20]. (6) none central obesity, characterized by low WHR (Male WHR < 0.90, Female WHR < 0.85). Finally, the overall HLS was calculated as the sum of the number of healthy lifestyle behaviors, ranging from 0 to 6, with a higher score indicating a healthier lifestyle.

### 2.3 Covariates

Following a standard protocol, anthropometric indicators including weight, height, systolic blood pressure (SBP), and diastolic blood pressure (DBP) were measured by well-trained investigators. Height and weight were measured by a standard scale, with the subjects wearing light clothing and no shoes. Blood pressure was measured in a sitting position with a mercury sphygmomanometer. SBP and DBP were recorded as the average of three repeat measurements within a 30 s interval. Whole-blood and serum samples were collected from each subject after an overnight fast. Biochemical variables (including total cholesterol (TC), triglyceride (TG), low-density lipoprotein cholesterol (LDL-C), high-density lipoprotein cholesterol (HDL-C), uric acid (UA)) were determined using a biochemical auto-analyzer (Hitachi 7060, Tokyo, Japan). Fasting plasma glucose (FPG) was determined using the glucose oxidase method with a Beckman Glucose Analyzer (Beckman Instruments, Irvine, CA, USA).

### 2.4 Outcome ascertainment

In our study, the outcome of FNCD refers to participants developing one of four chronic diseases (T2DM, cancer, stroke, and coronary heart disease) during the follow-up period. MCCs means that a person with FNCD status develops at least one of the other three diseases during the follow-up period, that is, two or more disease states co-exist.

The incidence of outcomes (T2DM, cancer, CVD including stroke and coronary heart disease) in the cohort was identified through the local chronic disease surveillance system, or by field epidemiological investigation [21]. The Zhejiang chronic disease surveillance system, which was established in 2001, was responsible for registering all the regional incidences of chronic diseases. These outcome events were diagnosed by certificated health practitioners (hospital staffs, community doctors). Administration professionals at the hospitals then checked these events and reported to the regional Center for Disease Control and Prevention (CDC) via the online system. The regional CDC verifies the events and removes duplicate records using the identification numbers. Data on the incidence of diabetes, coronary heart disease, and stroke were also obtained from field epidemiological surveys. The interviewer asked each subject if they had been diagnosed by a clinician. The occurrence or cause of the events is classified according to the International Classification of Diseases, 10th edition (ICD-10). When modeling the risk of FNCD, the end point of follow-up was to the date of diagnosis of the first NCD, or the endpoint of follow-up. When modeling the risk of the progression of FNCD to MCCs and MCCs, the end point of follow-up was considered to be the date of a subsequent second NCD, or the endpoint of follow-up, whichever occurred first.

### 2.5 Statistics

The participants were stratified into four groups based on the levels of HLS as ≤ 2, 3, 4 and ≥5, respectively. Continuous variables were described as means and standard deviations (SD) for normal distribution and median (interquartile range) with skewed distribution. Categorical variables were expressed as numbers (percentages). For comparisons among the four groups, one-way ANOVA was applied for continuous variables, and the chi-square test or Fisher exact test for categorical variables.

We used the Cox proportional hazards model to estimate the hazard ratios (HRs) and 95% CIs of HLS with the incidence of FNCD, the progression of FNCD to MCCs, and MCCs with ≤2 HLS as reference group. Two models were built. In Model 1, we adjusted for age and gender. In Model 2 we further adjusted for the area of residents, family history of NCDs, the intake of antihypertensive and lipid-lowering drugs.

Several sensitivity analyses for the Cox proportional hazards analyses were conducted. Firstly, we estimated the association between six individual lifestyles and the risk of FNCD. Second, we also analyzed the relationship between HLS and the risk of FNCD stratified by age (≤55 years vs. >55 years) and gender (female vs. male). Third, we evaluated the association of the combined HLS with different types of MCCs.

We selected the available metabolic biomarkers from the Zhejiang cohort for the mediation analyses based on knowledge of potential causal pathways that may contribute to the development of chronic diseases [[22], [23], [24]]. Some studies have found that lifestyle influences diseases by mediating metabolic components. Lu Q, Chen J et al., and Si J, Li J et al., discovered the mediating role of lipid metabolites in the relationship between a healthy lifestyle and the risk of CVD [25,26]. Thorburn AN et al. highlighted the role of diet, metabolites, and their associated immune pathways in the development of inflammatory diseases [27]. Assi N et al. found that relevant metabolic characteristics were identified as mediating the relationship between lifestyle exposure and HCC risk [28].

The selected biomarkers were considered as potential mediators following a two-step analysis. First, we assessed the associations of all biomarkers with the healthy lifestyle score using multivariable-adjusted linear regression models. Second, we assessed the relationships between biomarkers that were significantly associated with the HLS and the risks of outcomes using the multivariable-adjusted Cox regression model. Then we used the mediation package in R to examine whether the associations between HLS with the incidence of FNCD and MCCs were intermediated by the level of selected biochemical variables [29,30]. Indirect, direct, and total effects for each mediator were computed by combining the mediator and outcome models with the adjustment of all the covariates in Model 2. Nonparametric boot-strap resampling was used to compute the CIs of the proportions of mediations.

Furthermore, we calculated the population-attributable fractions (PAF) using the formula: $\sum_{i=0}^{k}{pd}_{i}\left(\frac{{HR}_{i}-1}{{HR}_{i}}\right)=1-\sum_{i=0}^{k}\frac{{pd}_{i}}{{HR}_{i}}$

(*pd_i_* = proportion of cases falling into *i*th exposure level; *HR_i_* = relative risk comparing *i*th exposure level with unexposed group [*i* = 0]) to estimate the proportion of FNCD, FNCD to MCCs, and MCCs that could theoretically be avoided if participants adhered to HLS > 3 [31]. In our analysis, we used HLS > 3 as the unexposed group, and HLS ≤ 3 as the exposure levels. All of the statistical analyses were performed using R software version 4.2.2. The two-sided P-value < 0.05 was defined as statistically significant.

## 3 Results

### 3.1 Baseline demographic data

The baseline demographic data of the study population is presented in Table 1. 43.1% of the subjects were males with the mean age of 55.5 years (SD = 13.5 years). 68.9% of participants were none-smokers and 76.9% were none-to-moderate alcohol drinkers. 974 subjects (9.2%) kept a diet with score 0, 728 people kept a diet with score 3 (6.9%) and the majority of people kept a diet with score 1−2. The exercise intensity was significantly higher in groups with a greater number of healthy lifestyle behaviors, for example, 255.5 (133.7−365.4) met-h/w in the group with HLS ≥5 versus 156.8 (111.2−276.5) met-h/w in the group with HLS ≤2. The baseline data of BMI and WHR were significantly higher in the group with lower HLS, indicating that weight management was more difficult in the group of people with worse lifestyle behaviors. There was a significant distribution difference between men and women in lifestyle choices and women were inclined to maintain a healthier lifestyle. 1119 (18.6%) female subjects had HLS ≥5, and 1129 (18.8%) had HLS ≤2 while male subjects had a higher proportion of HLS ≤2 (42.1%) and low of HLS ≥5 (8.4%). The family history of chronic diseases in each group was not significantly different.

**Table 1:** The baseline information of the study cohort according to the healthy lifestyle score (HLS) category.

			HLS		
Variants	Total	≤2	3	4	≥5
n (%)	10566	3047 (28.8)	3198 (30.3)	2817 (26.7)	1504 (14.2)
Gender					
Male (n, %)	4559 (43.1)	1918 (42.1)	1336 (29.3)	920 (20.2)	385 (8.4)
Female (n, %)	6007 (56.9)	1129 (18.8)	1862 (31.0)	1897 (31.6)	1119 (18.6)
Age (x¯ ± sd)	55.5 ± 13.5	58.5 ± 12.1	56.8 ± 13.2	53.9 ± 14.1	49.7 ± 13.6
Family History (n, %)					
Hypertension	2766 (26.2)	773 (25.4)	820 (25.6)	757 (26.9)	416 (27.7)
T2DM	477 (4.51)	132 (4.33)	126 (3.94)	140(4.97)	79 (5.25)
Stroke	326 (3.09)	108 (3.54)	84 (2.63)	91 (3.23)	43 (2.86)
CHD	277 (2.62)	75 (2.46)	83 (2.60)	77 (2.73)	42 (2.79)
Smoking (n, %)	3286 (31.1)	1786 (58.6)	948 (29.6)	479 (17.0)	73 (4.9)
Alcohol intake ≤1−2 times/w (n, %)	8125 (76.9)	1522 (50.0)	2541 (79.5)	2577 (91.5)	1485 (98.7)
Diet score n (n, %)					
0	974 (9.2)	413 (13.6)	296 (9.3)	214 (7.6)	51 (3.4)
1	5186 (49.1)	2159 (70.9)	1702 (53.2)	1121 (39.8)	204 (13.6)
2	3678 (34.8)	415 (13.6)	996 (31.1)	1224 (43.5)	1043 (69.3)
3	728 (6.9)	60 (2.0)	204 (6.4)	258 (9.2)	206 (13.7)
Physical Activity (met-h/w) median (p25 - p75)	178.5 (114.1−302.4)	156.8 (111.2−276.5)	166.2 (111.0−300.8)	187.3 (116.2−303.8)	255.5 (133.7−365.4)
BMI (kg/m^2^) (x¯ ± sd)	23.2 ± 3.3	24.8 ± 3.5	23.4 ± 3.5	22.1 ± 2.6	21.5 ± 1.9
WHR (x¯ ± sd)	0.87 ± 0.07	0.92 ± 0.06	0.88 ± 0.06	0.85 ± 0.06	0.81 ± 0.05
TC (mmol/L) (x¯ ± sd)	4.47 ± 1.06	4.62 ± 1.06	4.50 ± 1.06	4.37 ± 1.06	4.26 ± 1.03
TG (mmol/L) median (p25 - p75)	1.20 (0.85−1.77)	1.38 (0.97−2.01)	1.24 (0.86−1.80)	1.11 (0.81−1.62)	1.01 (0.75−1.40)
HDL-C (mmol/L) (x¯ ± sd)	1.34 ± 0.33	1.31 ± 0.34	1.35 ± 0.34	1.36 ± 0.32	1.38 ± 0.31
LDL-C (mmol/L) (x¯ ± sd)	2.86 ± 0.78	2.97 ± 0.80	2.88 ± 0.77	2.79 ± 0.77	2.74 ± 0.71
FPG (mmol/L) (x¯ ± sd)	4.95 ± 0.71	5.01 ± 0.73	4.96 ± 0.71	4.92 ± 0.69	4.89 ± 0.68
UA (μmol/L) (x¯ ± sd)	321.6 ± 89.7	357.4 ± 93.8	320.2 ± 84.7	300.6 ± 81.5	284.5 ± 77.1
**Morbidity of NCDs**					
Cancer (n, %)	601 (5.69)	205 (6.73)	185 (5.78)	145 (5.15)	66 (4.39)
T2DM (n, %)	508 (4.81)	208 (6.83)	172 (5.38)	92 (3.27)	36 (2.39)
CVD (n, %)	613 (5.80)	229 (7.52)	204 (6.38)	127 (4.51)	53 (3.52)
FNCD (n, %)	1572 (14.9)	570 (18.7)	514 (16.1)	341 (12.1)	147 (9.8)
MCCs (n, %)	149 (1.41)	73 (2.4)	46 (1.4)	23 (0.8)	7 (0.5)

HLS: Healthy Lifestyle Score.

CHD: coronary heart disease.

CVD: cardiovascular disease.

NCDs: non-communicable diseases include cancers, CVD (stroke and CHD) and T2DM.

FNCD: First Non-Communicable Chronic Disease.

MCCs: multiple chronic conditions, at least two kinds of non-communicable diseases.

MCCs: multiple chronic conditions, at least two kinds of non-communicable diseases.

### 3.2 The associations between HLS and the risk of FNCD and its progression to MCCs

During 97841.34 person-years of follow-up (median 9.26 years), 1572 participants (14.9%) developed FNCD. After an overall median follow-up time of 9.92 years, 601 participants (5.69%) developed cancer, 508 participants (4.81%) developed T2DM, 613 participants (5.80%) developed CVD and 149 participants (1.4%) developed two or more chronic diseases (defined as MCCs). The multimorbidity pattern of NCDs is presented in Supplementary Table S1. The most common multimorbidity pattern was CVD and T2DM (63 events) among participants with a crude incidence rate equal to 6 events per 10,000 person-years. Similar common patterns were CVD and cancers (40 events) or cancers and T2DM (31 events).

Table 2 presents the risk of FNCD, progression from FNCD to MCCs, and MCCs regarding the HLS groups. HLS was inversely associated with the risk of FNCD. With the subjects with HLS ≤2 as the reference and adjustment for potential confounding factors, HRs (95% CI) for the risk of FNCD were 0.89 (0.79, 1.00), 0.72 (0.63, 0.82), 0.68 (0.56, 0.82) in the subjects with HLS of 3, 4, and ≥ 5, respectively. Significant trends were found with each score added to HLS.

**Table 2:** The associations between HLS and the morbidity of FNCD, progression from FNCD to MCCs, the morbidity of MCCs.

HLS	Number of events	Person years	Incidence (%)	HR (95%CI)*	*P*-value^a^	HR (95%CI)^b^	*P*-value^b^	PAF (%)^b^
Baseline to FNCD								
≤2	570	27422.50	2.08	1.0 (ref.)	–	1.0 (ref.)	–	–
3	514	29330.55	1.75	0.89 (0.79, 1.00)	0.054	0.89 (0.79, 1.00)	0.062	–
4	341	26509.28	1.29	0.72 (0.62, 0.82)	<0.001	0.72 (0.63, 0.82)	<0.001	17.5 (11.2, 23.7)
≥5	147	14579.01	1.01	0.68 (0.56, 0.82)	<0.001	0.68 (0.56, 0.82)	<0.001	
*P* for trend					<0.001		<0.001	
FNCD to MCCs								
≤2	73	1874.81	3.89	1.0 (ref.)	–	1.0 (ref.)	–	–
3	46	1902.68	2.42	0.65 (0.44, 0.94)	0.022	0.65 (0.45, 0.96)	0.028	–
4	23	1333.34	1.72	0.46 (0.29, 0.75)	0.0017	0.47 (0.29, 0.76)	0.0021	37.0 (15.5, 56.3)
≥5	7	559.67	1.25	0.39 (0.18, 0.85)	0.018	0.39 (0.18, 0.85)	0.018	
*P* for trend					<0.001		<0.001	
Baseline to MCCs								
≤2	73	29297.31	0.25	1.0 (ref.)	–	1.0 (ref.)	–	–
3	46	31233.23	0.15	0.62 (0.43, 0.90)	0.012	0.63 (0.43, 0.92)	0.015	–
4	23	27844.62	0.083	0.39 (0.24, 0.63)	<0.001	0.40 (0.25, 0.65)	<0.001	42.9 (23.4, 61.0)
≥5	7	15138.68	0.046	0.31 (0.14, 0.67)	0.003	0.31 (0.14, 0.69)	0.004	
*P* for trend					<0.001		<0.001	

FNCD: First Non-Communicable Chronic Disease.

MCCs: multiple chronic conditions, at least two kinds of non-communicable diseases.

a Adjustment for age and gender.

b Adjustment for age, gender, family history, area, the intake of antihypertensives and lipid-lowering drugs.

Similar results were also found in the progression from FNCD to MCCs and the morbidity of MCCs. HRs (95% CI) for the risk of progression from FNCD to MCCs were 0.65 (0.45, 0.96), 0.47 (0.29, 0.76), 0.39 (0.18, 0.85) in the subjects within the groups of HLS = 3, = 4 and ≥ 5. Subjects with HLS ≥ 5 had 61% (15%, 82%) reduced risk of FNCD progression to MCCs. HRs (95% CI) for the risk of MCCs were 0.63 (0.43, 0.92), 0.40 (0.25, 0.65), 0.31 (0.14, 0.69) in the participants with HLS of 3, 4, and ≥ 5.

HRs significantly decreased as the number of healthy lifestyle factors increased in stratified groups (Supplementary Table S2). For the group with HLS = 3, = 4, and ≥5, male HRs (95% CI) were respectively 0.78 (0.65, 0.92), 0.61 (0.49, 0.76), and 0.71 (0.51, 0.98), female HRs (95% CI) were respectively 1.01 (0.85, 1.20), 0.80 (0.66, 0.96), 0.68 (0.53, 0.86). Males were less likely to acquire FNCD when HLS reached 3, and females benefited significantly when HLS reached 4. When stratified the patient cohort by age (≤ 55 or >55 years at recruitment), in both the younger and the older group, the adherence to healthier lifestyles had a negative correlation with FNCD. For the older group with age over 55 years, HRs (95% CI) were respectively 0.87 (0.76, 1.01), 0.68 (0.58, 0.80), and 0.62 (0.49, 0.79) with each HLS group increase. For the younger group, the decrease in HRs were more obvious when HLS got larger. The respective HRs (95% CI) were 0.86 (0.68, 1.10), 0.64(0.50, 0.83), and 0.51 (0.37, 0.70) for the younger group.

### 3.3 Population attributable fraction of unhealthy lifestyle

The results of PAFs indicated that if the subjects adhered to >3 healthy lifestyle factors, they could theoretically avoid 17.5% (11.2%, 23.7%) getting FNCD and 42.9% (23.4%, 61.0%) of getting MCCs (Table 2). When further investigating the influence of lifestyle factors on the progression from FNCD to MCCs, if the subjects adhered to more than three healthy lifestyle behaviors, they were less likely to progress to MCCs after diagnosed with FNCD. For those who adhere to HLS > 3, they could theoretically prevent the occurrence of more chronic diseases by 37.0% (15.5%, 56.3%). Based on the stratified analysis between HLS and FNCD (Supplementary Table S2), females could avoid 14.8% (7.85%, 22.1%) and males could avoid 23.8% (12.3%, 34.8%) of getting FNCD. The younger group could theoretically avoid 21.9% (12.7%, 31.2%) and the older group could avoid 20.9% (13.3%, 28.5%) to get FNCD. CVD and T2DM were the major pattern of MCCs and 64.7% (39.7%, 85.9%) of getting both could be avoided if ≥ 4 low-risk lifestyle behaviors were maintained.

### 3.4 The mediation role of metabolic components

The associations between the metabolic components and outcomes were shown in Table 3. Six metabolic components were detected in the associations of HLS with the risk of FNCD and MCCs. Both the relationship of HLS with the risk of FNCD and HLS with the risk of MCCs were potentially mediated by TC, TG, LDL-C, HDL-C, FPG, and UA. The proportion of mediation effect ranged from 5.02% (TC) to 22.2% (TG) regarding the risk of FNCD, from 5.94% (LDL-C) to 20.1% (TG) regarding the risk of MCCs.

**Table 3:** The mediation of metabolic components in the roles of HLS on FNCD and MCCs.

	Total effect		Direct effect		Indirect effect		Proportion of mediation	
	beta (95%CI)	*P*	beta (95%CI)	*P*	beta (95%CI)	*P*	% (95%CI)	*P*
From baseline to FNCD								
TC (mmol/L)	−0.020 (−0.028, −0.012)	<0.001	−0.019 (−0.027, −0.011)	<0.001	−0.0010 (−0.0019, −0.00013)	0.024	5.02 (0.84, 10.9)	0.024
TG (mmol/L)	−0.020 (−0.028, −0.012)	<0.001	−0.016 (−0.023, −0.0081)	<0.001	−0.0045 (−0.0061, −0.0030)	<0.001	22.2 (13.4, 38.0)	<0.001
LDL-C (mmol/L)	−0.020 (−0.028, −0.012)	<0.001	−0.019 (−0.027, −0.011)	<0.001	−0.0010 (−0.0018, −0.00025)	0.008	5.12 (1.23, 10.7)	0.008
HDL-C (mmol/L)	−0.020 (−0.028, −0.012)	<0.001	−0.018 (−0.026, −0.010)	<0.001	−0.0017 (−0.0026, −0.00097)	<0.001	8.46 (4.68, 15.0)	<0.001
FPG (mmol/L)	−0.019 (−0.027, −0.011)	<0.001	−0.018 (−0.025, −0.0098)	<0.001	−0.0017 (−0.0025, −0.00089)	<0.001	8.55 (4.50, 15.7)	<0.001
UA^a^ (μmol/L)	−0.019 (−0.028, −0.010)	<0.001	−0.017 (−0.026, −0.0084)	<0.001	−0.0021 (−0.0036, −0.00062)	0.008	10.8 (3.05, 21.9)	0.008
From baseline to MCCs								
TC (mmol/L)	−0.0082 (−0.016, −0.0032)	<0.001	−0.0077 (−0.015, −0.0028)	<0.001	−0.00052 (−0.0010, −0.00005)	0.036	6.30 (0.60, 15.09)	0.036
TG (mmol/L)	−0.0084 (−0.016, −0.0033)	<0.001	−0.0067 (−0.014, −0.0019)	<0.001	−0.0017 (−0.0026, −0.00089)	<0.001	20.1 (10.3, 41.8)	<0.001
LDL-C (mmol/L)	−0.0081 (−0.015, −0.0031)	<0.001	−0.0076 (−0.015, −0.0027)	<0.001	−0.00048 (−0.00091, −0.00007)	0.028	5.94 (0.81, 14.3)	0.028
HDL-C (mmol/L)	−0.0081 (−0.015, −0.0030)	<0.001	−0.0074 (−0.015, −0.0026)	<0.001	−0.00067 (−0.0011, −0.0003)	<0.001	8.33 (3.70, 17.8)	<0.001
FPG (mmol/L)	−0.0074 (−0.014, −0.0027)	<0.001	−0.0069 (−0.013, −0.0023)	<0.001	−0.00049 (−0.00082, −0.00023)	<0.001	6.65 (3.03, 14.5)	<0.001
UA^a^ (μmol/L)	−0.0082 (−0.016, −0.0031)	<0.001	−0.0076 (−0.015, −0.0026)	<0.001	−0.00058 (−0.0014, 0.0001)	0.098	7.07 (−1.26, 19.3)	0.098

Note: adjustment for age, gender, area, family history, the intake of antihypertensives and lipid-lowering drugs.

Before statistical analysis, the values of TC, TG, LDL-C, HDL-C, FPG and UA were nature log-transformed.

a Among the whole study population, only 9898 subjects had the value of uric acid and we take these data into analysis.

## 4 Discussion

Although the associations between lifestyles and certain kinds of NCDs have been verified in previous studies, we noted that the population-based cohort studies focusing on the influence of overall lifestyles on multimorbidity of chronic diseases were limited. In this prospective Zhejiang cohort, 1.4% participants developed MCCs during the follow up. The relatively low incidence in the context of MCC was partly due to the site selection (Zhejiang is one of the most developed and educated areas in the southeast coast of China with adequate medical resources and nice ecological environment). Adherence to a greater number of healthy lifestyle behaviors was found to be associated with a lower risk of getting FNCD and progression to MCCs, namely type 2 diabetes, cancer, and cardiovascular disease. There was a homogeneity in the associations, between the number of healthy lifestyles and the incidence of MCCs, regardless of how many NCDs were diagnosed. For each number increment in the healthy lifestyle behavior, there was a lower risk of developing NCDs. There was a substantially higher incidence rate among patients with low HLS and the highest overall incidence rate of FNCD and MCCs was in the subgroup of HLS = 0. The elevation of HLS from 0 to 1 resulted in a 350/100,000 decrease of MCCs incidence. When HLS reached four, the incidence of FNCD and MCCs both dramatically decreased.

Findings were quite consistent in FNCD analyses where the subjects were stratified by gender or age. Subjects who adhere to HLS > 2 were less likely to progress to MCCs after being diagnosed with FNCD. Women were more likely to persist in a healthier lifestyle but males benefited more from a healthy lifestyle than females. The maintenance of more low-risk lifestyle factors could theoretically prevent the occurrence of FNCD and MCCs, especially non-malignant chronic conditions including CVD and T2DM. High-risk life behaviors have been proven to have causative effects on chronic metabolic diseases. For example, obesity and diabetes are interdependent through insulin resistance, smoking greatly increases the risk of CVD by vascular endothelial injury [[32], [33], [34]]. Avoiding these harmful lifestyle behaviors might have a protective effect on the circulation system and metabolism. For example, smoking cessation has been proved to be associated with decreased CVD incidence and all-cause mortality among T2DM patients [35].

The metabolic components might have explained a proportion of the associations between the overall HLS with FNCD and MCCs. TG and UA were two major metabolic components found to play important roles in the associations between HLS and disease conditions. McGarry proposed the concept of glycolipid metabolism in diabetes mellitus in 2002, suggesting that excessive deposition of circulating free fatty acids (FFAs) and TGs are key factors for the regulation of insulin action [36]. TG is an important biomarker of dyslipidemia with adverse outcomes in patients with hypertriglyceridemia (HTG) and elevated TGs are common in T2DM and contribute to a two-fold increase in atherosclerotic cardiovascular disease (ASCVD) in T2DM [37]. UA is a major enzyme for allantoin conversion and an increasing level of UA can stimulate metabolic imbalance and contribute to diabetes and several kinds of cancers [38]. Recent data reveals that serum uric acid level is positively associated with an increased incidence of T2DM among females [39]. Our findings confirmed the potential mediating role of TG and UA in the multimorbidity of chronic diseases.

The unique strength of our study includes: (1) We explored the influence of combined healthy lifestyle behaviors on the risk of first NCD, its progression to MCCs, and finally the morbidity of MCCs in the Chinese population; (2) Our study included a long period of follow-up, and an extensive collection of data on clinical biomarkers, which allowed us to comprehensively evaluate the potential mechanisms underlying the observed associations.

However, there are several limitations in this study. Firstly, we only used lifestyle information at the baseline, without considering potential lifestyle changes during the follow-up period. Accordingly, the present study might underestimate the significance of the combined effects of lifestyle factors on the risks of FNCD, MCCs and the progression from FNCD to MCCs. Secondly, there was a lack of treatment data following FNCD. To our knowledge, some medications have protective effects in terms of NCDs, while some are quite detrimental. Metformin, for example, is widely recommended as the first-line therapy for most T2DM patients. Metformin has been found to have protective effects on lipid control, cardiovascular events, and possibly some cancers, and it may have a positive effect on extending lifespan and might have an overestimate in the correlations between HLS and MCCs [[40], [41], [42]]. In contrast, cancer therapy is well recognized for increased CHD and might have resulted in an underestimation [43]. In our statistical models, we adjusted for the intake of antihypertensives and lipid-lowering drugs to lower the influences of medications. Nevertheless, our observed associations should not be affected by treatment data for it is independent of lifestyle. Thirdly, the exclusion criteria for participants might introduce selection bias. However, because of the large sample size, the absolute difference between the included and excluded populations was minimal, resulting in a small selection bias. Besides, there was still a possibility of residual confounding by additional unmeasured or unknown factors, particularly socioeconomic status. However, this was a community cohort study with participants of similar socioeconomic status, which had little impact on the findings. Finally, the absolute risks associated with lifestyle factors heavily depend on the underlying habits of the study population. We generated the association between healthy lifestyles and three transitions of NCDs based on the Zhejiang cohort, which however may not reflect the whole picture of the general Chinese population because of the diversity of diet and life habits.

In conclusion, our findings suggested that adherence to overall healthy lifestyle behaviors was associated with a lower risk of getting multiple chronic diseases. Our findings support the importance of public health initiatives and interventions targeting improving health behaviors in combination to ameliorate the risk of chronic diseases and subsequently lower the burden of disease management. Further studies with a larger cohort monitoring of dynamic lifestyle changes are needed to validate our findings.

## Disclosure of interest

None.
