# Supplementary material for: Healthy lifestyle decreases the risk of the first incidence of non-communicable chronic disease and its progression to multimorbidity and its mediating roles of metabolic components: a prospective cohort study in China
Source: J Nutr Health Aging. 2024 Feb 1;28(3):100164. doi: 10.1016/j.jnha.2024.100164 (PMC12880506; doi:10.1016/j.jnha.2024.100164)
Supplement: Supplementary file 1 [file mmc1.docx]

| **Supplementary table 1. The associations between HLS and the morbidity of different types of MCCs** | | | | | | | | |
| --- | --- | --- | --- | --- | --- | --- | --- | --- |
| **HLS** | **Cases** | **Person years** | **Incidence（%）** | **HR (95%CI)*** | ***P*-value*** | **HR (95%CI)**** | ***P*-value**** | **PAF (%)**** |
| **CVD and T2DM** | | | | | | | | |
| ≤3 | 55 | 60530.54 | 0.091 | 1.0 (ref.) | - | 1.0 (ref.) | - | - |
| ≥4 | 8 | 42983.30 | 0.019 | 0.26 (0.12, 0.54) | <0.001 | 0.26 (0.12, 0.55) | <0.001 | 64.7 (39.7, 85.9) |
| **CVD and cancers** | | | | | | | | |
| ≤3 | 29 | 60530.54 | 0.048 | 1.0 (ref.) | - | 1.0 (ref.) | - | - |
| ≥4 | 11 | 42983.30 | 0.026 | 0.83 (0.41, 1.68) | 0.61 | 0.85 (0.42, 1.72) | 0.65 | 11.1 (-35.2, 53.7) |
| **Cancers and T2DM** | | | | | | | | |
| ≤3 | 23 | 60530.54 | 0.038 | 1.0 (ref.) | - | 1.0 (ref.) | - | - |
| ≥4 | 8 | 42983.30 | 0.019 | 0.56 (0.25, 1.28) | 0.17 | 0.59 (0.26, 1.34) | 0.20 | 30.8 (-16.8, 72.3) |

CVD include stroke and CHD

MCCs: multiple chronic conditions, including at least two kinds of non-communicable diseases

* Adjustment for Cox-regression adjustments: age and gender

** Adjustment for age, gender, area, family history, the intake of depressor and lipid-lowering drugs

| **Supplementary table 2. The associations between HLS and FNCD in the cohort** **when stratified by gender and age** | | | | | | | | |
| --- | --- | --- | --- | --- | --- | --- | --- | --- |
| **HLS** | **Cases** | **Person years** | **Incidence（%）** | ***HR*(95%CI)*** | ***P*-value*** | ***HR*(95%CI)**** | ***P*-value**** | **PAF (%)**** |
| **Sex** |  |  |  |  |  |  |  |  |
| **Male** |  |  |  |  |  |  |  |  |
| ≤2 | 364 | 17225.50 | 2.11 | 1.0 (ref.) | **-** | 1.0 (ref.) | - | - |
| 3 | 197 | 12301.37 | 1.60 | 0.78 (0.66, 0.93) | 0.0049 | 0.78 (0.65, 0.92) | 0.0042 | - |
| 4 | 102 | 8605.51 | 1.19 | 0.62 (0.50, 0.78) | <0.001 | 0.61 (0.49, 0.76) | <0.001 | 23.8 (12.3, 34.8) |
| ≥5 | 41 | 3643.17 | 1.13 | 0.73 (0.53, 1.01) | 0.055 | 0.71 (0.51, 0.98) | 0.039 |  |
| *P* for trend |  |  |  |  | <0.001 |  | <0.001 |  |
| **Female** |  |  |  |  |  |  |  |  |
| ≤2 | 206 | 10197 | 2.02 | 1.0 (ref.) | **-** | 1.0 (ref.) | - | - |
| 3 | 317 | 17029.18 | 1.86 | 1.01 (0.85, 1.20) | 0.92 | 1.01 (0.85, 1.20) | 0.92 | - |
| 4 | 239 | 17903.77 | 1.33 | 0.80 (0.66, 0.96) | 0.017 | 0.80 (0.66, 0.96) | 0.019 | 14.8 (7.85, 22.1) |
| ≥5 | 106 | 10935.84 | 0.97 | 0.67 (0.53, 0.86) | 0.001 | 0.68 (0.53, 0.86) | 0.0015 |  |
| *P* for trend |  |  |  |  | <0.001 |  | <0.001 |  |
| **Age** |  |  |  |  |  |  |  |  |
| **≤55 years** |  |  |  |  |  |  |  |  |
| ≤2 | 131 | 11660.03 | 1.12 | 1.0 (ref.) | **-** | 1.0 (ref.) | - |  |
| 3 | 148 | 13871.84 | 1.07 | 0.86 (0.68, 1.09) | 0.22 | 0.86 (0.68, 1.10) | 0.23 |  |
| 4 | 117 | 14272.74 | 0.82 | 0.63 (0.49, 0.82) | <0.001 | 0.64 (0.50, 0.83) | <0.001 | 21.9 (12.7, 31.2) |
| ≥5 | 64 | 9556.66 | 0.67 | 0.49 (0.36, 0.68) | <0.001 | 0.51 (0.37, 0.70) | <0.001 |  |
| *P* for trend |  |  |  |  | <0.001 |  | <0.001 |  |
| **>55 years** |  |  |  |  |  |  |  |  |
| ≤2 | 439 | 15762.47 | 2.79 | 1.0 (ref.) | **-** | 1.0 (ref.) | - |  |
| 3 | 366 | 15458.71 | 2.37 | 0.87 (0.75, 1.00) | 0.048 | 0.87 (0.76, 1.01) | 0.060 |  |
| 4 | 224 | 12236.54 | 1.83 | 0.67 (0.57, 0.79) | <0.001 | 0.68 (0.58, 0.80) | <0.001 | 20.9 (13.3, 28.5) |
| ≥5 | 83 | 5022.35 | 1.65 | 0.61 (0.48, 0.77) | <0.001 | 0.62 (0.49, 0.79) | <0.001 |  |
| *P* for trend |  |  |  |  | <0.001 |  | <0.001 |  |

When stratified by gender:

* adjustment for age, ** adjustment for age, area, family history, the intake of depressor and lipid-lowering drugs

When stratified by age:

* adjustment for gender,** adjustment for gender, area, family history, the intake of depressor and lipid-lowering drugs
